# Sizing of reactors by charts of Damköhler's number for solutions of dimensionless design equations

**DOI:** 10.1016/j.heliyon.2020.e05386

**Published:** 2020-11-02

**Authors:** Héctor L. Otálvaro-Marín, Fiderman Machuca-Martínez

**Affiliations:** a GAOX, Escuela de Ingeniería Química, Universidad del Valle, A.A. 25360, Cali, Colombia; b MADE Group, Food Engineering Program, Universidad de la Amazonia, Florencia, Colombia; c IDEI Group, I+D Educación e Ingeniería, Cali, Colombia

**Keywords:** Chemical engineering, Catalyst, Environmental chemical engineering, Chemical reaction engineering, Computer-aided engineering, Scale-up, Reactor design, Reaction engineering, Modeling, Kinetic rate

## Abstract

The reaction kinetic rate and mass transport play an important role in the sizing and scale-up of reactors. The Damköhler's dimensionless number (Da) is the quotient of these effects. A new interpretation of Da as a local property is introduced Da(x,y,z,t). A new graphical methodology is proposed for the sizing and scale-up of unidirectional flow reactors and CSTRs. The partial differential equation (PDE) and algebraic that describe the continuity within these reactors transform into dimensionless variables, and the conversion at the output is expressed as a function of the conditions at the input $Da_{0}$. The operating conditions as volumetric flow, residence time; design variables as reactor volume; and intrinsic reaction rate are involved in $Da_{0}$. The equations are solved numerically to develop the design charts $Da_{0}$ vs X.

The design volume is linear with $Da_{0}$, and the conversion is obtained from the charts ($Da_{0}$ vs X) or vice versa. Using these charts avoids the analytical or numerical solution of the PDE that governs the unidirectional flow reactors becoming an easy tool for scale-up. The article portrays how to use these diagrams. Reactors with $Da_{0}$< 0.1 have a low conversion per pass, the charts also allow estimating the number of recirculations required as a function of the overall conversion. Reactors with the same conversion have the same $Da_{0}$, both laboratory and industrial scale. Then, the Danumber is presented as a fundamental parameter for design and scaling-up these reactors.

## 1 Introduction

Dimensionless numbers play an important role in engineering application design. These numbers have a practical physical meaning based on transport phenomena (mass, heat, momentum), and their magnitude is a relative scale between phenomena. There are hundreds of dimensionless numbers, and some of their applications are: in fluid mechanics, the Reynolds (Re) and Froude (Fr) numbers are used in the design of turbofan engines [1] and studies of rainfall-runoff on dams [2], the Euler's number (Eu) is used for the design of hydrocyclones [3]; Rayleigh (Ra), Nusselt (Nu) and Prandtl (Pr) are used in the array design of insulating materials [4]; the Damköhler (Da), Péclet (Pe), Hatta (Ha) numbers, and others are used in the design of different types of reactors with heat and mass transfer [5, 6, 7].

There are two approaches to obtain dimensionless numbers from the dimensional analysis: classical dimensional analysis [8] and discriminated dimensional analysis. The latter discriminates the dimensions of space and the components of a variable, creating a greater number of variables and, by the Pi theorem, then a less quantity of dimensionless numbers is obtained. This approach has been used in free convection [9] and mixed convection [10] processes. An alternative way of obtaining dimensionless numbers is by non-dimensionalization of the differential equations that govern the phenomenon, using reference quantities that allow defining dimensionless variables [7].

Damköhler's numbers (Da and $Da_{II}$) are dimensionless numbers used to relate the intrinsic reaction rate to the mass transport rate [11]. Da is the ratio of the reaction rate to the global movement rate of the fluid (see supporting information S1). $Da_{II}$ is the ratio of reaction rate to diffusive transport across an interface in reaction with multiple phases. Péclet number (Pe) relates the global movement rate of the fluid to the diffusive mass transport rate. Pe and Da describe reactors where the diffusion, global movement, and reaction rate of the species take place simultaneously [12]. Different continuous reactive systems have indicated that the conversion (X) increases with Da[7, 13]. Simulated reactors with Dabetween 10^−5^ and 10^2^ showed the main aspects that control the conversion in membrane reactors [14, 15, 16, 17] and other continuous reactors with interfacial mass transport [18, 19].

**Figure 1:**
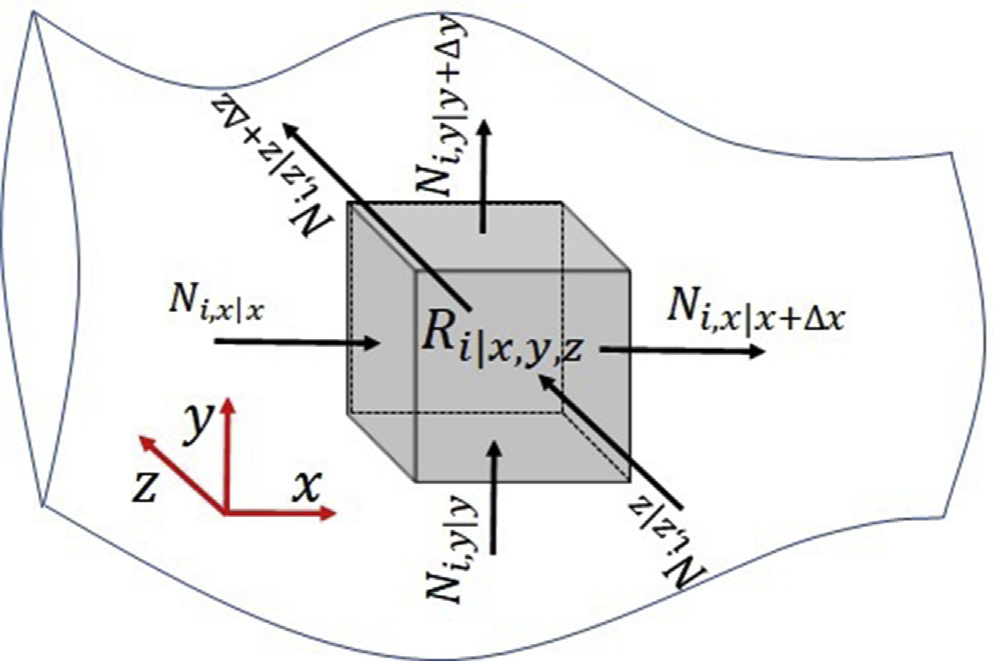
Schematic representation of any reactive system with mass transfer by diffusion, global movement, and reaction rate for a component i.

Continuous reactors of any scale present mass transport across the system boundaries while the reaction takes place simultaneously. The continuous stirred-tank reactor CSTR, ideally, does not have concentration gradients inside the reactor [20], but they present mass transport across their boundaries. In contrast, continuous unidirectional flow reactors such as tubular reactors, elongated flat plates, packed-bed reactors, plug flow reactors have concentration gradients, preferably in the direction of fluid flow. Each volume differential element has mass transport across its boundaries and a reaction local rate [21] (See Figure 1). The continuity equation of unidirectional flow reactors in the transient state is a partial differential equation PDE, which can be written in dimensionless terms.

Modeling and simulation based on Da were carried out in pilot-scale photocatalytic reactors of different geometries: fountain type [22], annular reactor [23], falling film, and tubular reactor with composite parabolic collectors CPCR [7]. An annular photocatalytic reactor within a batch/recycle system with a perfectly mixed tank was modeled as a function of Da and Pe [24, 25]. Therefore, Da is a parameter that can be estimated in different continuous reactive systems, geometries, scales, and recirculation systems. However, an intensive analysis of the Damköhler's number and the conversion, which generate tools for reactor scaling-up, have not been explicitly presented.

In this study, the derivation of Da from the continuity equation was developed. The conversion at the outlet of a CSTR and unidirectional flow reactors was modeled as a function of the Da. Simulations based on the numerical solution of the dimensionless algebraic and differential equations were carried out to construct the design chart of these reactors, grouping design and operation variables in the Da number. Different scenarios for the design and operation conditions, and conversion were analyzed for power-law kinetics.

## 2 Methodology

We will present the mathematical models that describe the unidirectional flow reactors and a CSTR as a function of dimensionless variables.

### 2.1 Effect of mass transport in continuous large reactors

Let us consider an infinitesimal volume within a reactor with mass flow as shown in Figure 1.

The continuity equation of the component A (i=A) in rectangular coordinates is given by Eq. (1). The derivation from a differential volume element was reported [21].

(1) $$\frac{\partial C_{A}}{\partial t}+\left(\frac{\partial N_{Ax}}{\partial x}+\frac{\partial N_{Ay}}{\partial y}+\frac{\partial N_{Az}}{\partial z}\right)=R_{A}$$

Where $C_{A}$ is the concentration of A-component (mol cm^−3^), t is the time (s), the first term at Eq. (1) is the change in local concentration over time, $N_{Ax},\;N_{Ay}$, $N_{Az}$ are the molar flux of A-component (mol cm^−2^ s^−1^) in the x, y, z components (cm) due to diffusion plus the global movement of the fluid (supporting information S1), the term inside parentheses is the change in concentration due to the mass transport by the motion of the bulk of the fluid and diffusion across the boundaries of the differential element, $R_{A}$ is the local intrinsic reaction rate of A (mol of A cm^−3^ s^−1^).

Consider the origin of the coordinate system located at the inlet of a reactor, as the fluid passes through the reactor increases the spatial coordinates. An analysis of Eq. (1) infers that all the terms are positive for a reaction when A is generated, and negative if A is consumed.

Solving the first term of Eq. (1), then, the change in concentration over time is equal to the reaction rate minus the change in concentration due to mass transport. Therefore, mass transport decreases the concentration change rate and reactor efficiency. This reduction in efficiency is noticeable in large enough reactors, particularly on a pilot or industrial scale. Thus, it is imperative to consider hydrodynamic and diffusive mass transport in the design of large-scale reactors and to avoid under-dimensioning the reactor [26]. The Damköhler number involves the phenomena of mass transport and reaction rate.

### 2.2 Damköhler number

We will present a modification to the Damköhler number according to Table 1, which will be more useful for the analyzes of this study.

**Table 1:** Modification of Da

Current approach	Proposed alternative (this work)
The Damköhler number is defined as a property of the reactor at the inlet.	The Damköhler number is defined as a local propertyDa(x,y,z,t).
$Da={-R_{A}}^{0}\tau_{R}/{C_{A}}^{0}$	$Da=-R_{A}\tau_{R}/{C_{A}}^{0}$
It is incorporated into the reaction rate	It is incorporated into the continuity equation
$-R_{A}=f\left(Da\right)$	$\frac{\partial C_{A}}{\partial t}=-\frac{\partial C_{A}}{\partial z}-Da$
Dimensional expression.	Dimensionless expression.

### 2.3 Da and Pe numbers at unidirectional flow reactors

**Figure 2:**
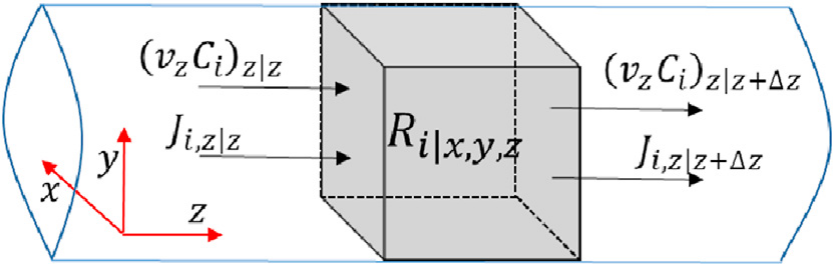
Scheme of a volume differential element within a unidirectional flow reactor with mass transfer by diffusion, global movement, and reaction rate.

The continuity equation for a unidirectional flow reactor (Figure 2) with length $L_{R}$on the z-axis, in a transient state, with constant density and constant diffusivity coefficient, is:

(2) $$\frac{\partial C_{A}}{\partial t}-D\frac{\partial^{2}C_{A}}{\partial z^{2}}+v\frac{\partial C_{A}}{\partial z}=R_{A}$$

This equation demonstrates how the diffusivity coefficient D(cm^2^ s^−1^), the average velocity on the z-axis v (cm s^−1^), and the kinetic rate $R_{A}$are related in the mass balance. The details of the derivation of Eq. (2) are found in the support information S1.

The first term is the concentration net-change rate within each differential element over time. The second term is the concentration change rate by a net diffusive transport (inlets and outlets) across the boundaries of each differential element. The third term is the concentration change rate by net hydrodynamic transport across the boundaries of each differential element, and $R_{A}$is the local reaction rate within each volume differential element.

The following expressions are defined:

(3) $$C_{A}\equiv C_{A}/{C_{A}}^{0}$$

(4) $$z\equiv z/L_{R}$$

(5) $$t\equiv t/\tau_{R}$$

where the bold variables are dimensionless; ${C_{A}}^{0}$is the initial concentration (mol cm^−3^); $L_{R}$ is the reactor length (cm); $\tau_{R}$ is the mean residence time inside the reactor (s); the mean residence time in a unidirectional flow reactor is $L_{R}/v$. Eq. (2) becomes:

(6) $$\frac{\partial C_{A}}{\partial t}=\frac{1}{Pe}\frac{\partial^{2}C_{A}}{\partial z^{2}}-\frac{\partial C_{A}}{\partial z}-Da$$

where:

(7) $$Da\equiv -R_{A}\tau_{R}/{C_{A}}^{0}$$

(8) $$Pe\equiv {L_{R}}^{2}/\left(D\tau_{R}\right)$$

Dais the Damköhler Number and Peis the Péclet Number. The negative sign in Eq. (7) was conveniently chosen to express positive the Dafor consumption reactions of A. Eq. (6) is the continuity equation of A as a function of dimensionless variables applied for any reaction rate.

Other equivalent Daand Peinterpretations are [13]:

(9) $$Da=\frac{-R_{A}L_{R}}{C_{A0}v}=\frac{-R_{A}V}{C_{A0}\dot{Q}}=\frac{-R_{A}V}{F_{A0}}=\frac{reaction\;rate}{\;hydrodynamic\;mass\;transport\;rate}$$

(10) $$Pe=\frac{L_{R}v}{D}=\frac{hydrodynamic\;mass\;transport\;rate}{\;diffusive\;mass\;transport\;rate}$$

Where Vis the reactor volume (cm^3^),$\;\dot{Q}$ is the volumetric flow (cm^3^ s^−1^) and $F_{A0}$is the molar flow at the inlet (mol s^−1^). Dacan be interpreted: (i) as a function of the product of the reaction rate and the mean residence time, or equivalently, (ii) as the quotient of a reaction rate between a hydrodynamic mass transport rate. Similarly, 1/Pe is (i) a function of the product of the diffusion coefficient by the mean residence time, or (ii) the ratio of the diffusive mass transport between the hydrodynamic mass transport [11].

Dais a function of the reaction rate Da = f($R_{A}$), therefore, of variables such as $C_{A}$, z-coordinate, time, and even concentration of other species $C_{i\neq A}$. In this work, we define Damköhler as a function f(x, y, z, t), contrary to the literature where Da is defined to the inlet conditions.

### 2.4 Unidirectional flow reactors with a power-law kinetic rate

Let us consider a reaction that follows an n-th power reaction rate, $-R_{A}=k{C_{A}}^{n}$, where k is the kinetic constant. Eq. (6) becomes (see supporting information S2):

(11) $$\frac{\partial C_{A}}{\partial t}=\frac{1}{Pe}\frac{\partial^{2}C_{A}}{\partial z^{2}}-\frac{\partial C_{A}}{\partial z}-{C_{A}}^{n}Da_{0}$$

where $Da_{0}$is a constant value obtained by evaluating Eq. (7) to the conditions at the inlet. Eq. (11) is specifically for systems that obey a reaction rate expressed by an n-th power law. This equation can be solved to determine the conversion at the reactor outlet as a function of $Da_{0}$.

#### 2.4.1 Boundary conditions

If the hydrodynamic mass transport is much higher than the diffusion mass transport, the term diffusive can be neglected. So, only one boundary condition is required at the reactor inlet. This condition is function of the operation conditions of the reactor according to Table 2.

**Table 2:** Boundary conditions at unidirectional flow reactors.

Boundary condition	Expression	Details
I. inlet concentration is constant	${\frac{dC_{A}}{dt}|}_{\left(z=0\right)}=0$	Use for a single-pass of the fluid through the reactor, the solution plots the profile of $C_{A}$in z in the transient state.
II. External recirculation with a mixing tank	${\frac{dC_{A}}{dt}|}_{\left(z=0\right)}=\frac{1}{\tau_{\mathrm{TK}}}\left({C_{A}}_{\left(z=1\right)}-{C_{A}}_{\left(z=0\right)}\right)$	External recirculation with a perfectly mixed tank without reaction and average residence time$\tau_{\mathrm{TK}}$. For $\tau_{\mathrm{TK}}=1$the mean residence time of the external tank and reactor are equal. Multiple passes through the reactor can be analyzed.
III. Immediate recirculation	${\frac{dC_{A}}{dt}|}_{\left(z=0\right)}={\frac{dC_{A}}{dt}|}_{\left(z=1\right)}={-\frac{\partial C_{A}}{\partial z}|}_{\left(z=1\right)}-{Da|}_{\left(z=1\right)}$	The reactor outlet is connected to the reactor inlet. Mathematically, it is as if two reactors were joined, and the change in the output of one is equal to the change in the input of the next. Multiple passes through the reactor can be analyzed.

### 2.5 Damköhler (Da) number at CSTR

The dimensionless modeling of a steady-state CSTR is easy to solve analytically and provides a quick estimate of the conversion per pass through the reactor from a given *Da* at the inlet. The mass balance of a steady-state CSTR is:

(12) $$F_{A}-F_{A0}=R_{A}V$$

where $F_{A0}$and $F_{A}$ are the molar flows of A (mol s^−1^) at the inlet and outlet respectively, and Vis the CSTR volume (cm^3^). Taking into account that $F_{A}=C_{A}\dot{Q}$, and considering a constant volumetric flow $\dot{Q}$, then, Eq. (12) can be rewritten as:

(13) $$C_{A}-C_{A0}=R_{A}\;\tau_{R}$$

where $\tau_{R}=V/\dot{Q}$. Considering a reaction that follows a power reaction rate, $R_{A}=-k{C_{A}}^{n}$ and using $C_{A}$= ${C_{A}}^{0}\left(1-X\right)$, then, Eq. (13) is rewritten as:

(14) $$\frac{X}{{\left(1-X\right)}^{n}}=\frac{k{C_{A0}}^{n}\;\tau_{R}}{C_{A0}}$$

Notice that the right side of this equation is $Da_{0}$, the Damköhler number (Eq. (7)) evaluated at the inlet of the CSTR. Frequently, other authors use this definition for Da[23, 27]; however, we want to differentiate the notation of Daand $Da_{0}$. This equation is highly useful since it relates the conversion at the reactor outlet with the Da at the inlet:

(15) $$Da_{0}=\frac{X}{{\left(1-X\right)}^{n}}$$

This result is consistent with the literature [28] for CSTR with $Da_{0}=k{C_{A0}}^{n-1}V/\dot{Q}$.

### 2.6 Design of a differential batch reactor

The general design equation, Eq. (1), applies to perfectly mixed and small enough batch reactors (differential reactors). These reactors are used on a laboratory scale to adjust kinetic parameters. Eq. (1) becomes:

(16) $$\frac{dC_{A}}{dt}=R_{A}$$

Applying the transformation to dimensionless variables:

(17) $$\frac{dC_{A}}{dt}=\frac{R_{A}\tau_{R}}{{C_{A}}^{0}}=-Da$$

The dimensionless expression is not useful for adjusting kinetics. However, for simulation and design, these reactors are easily described in their dimensionless expression by the Damköhler number.

For a power-law kinetics $R_{i}=k{C_{i}}^{n}$, Eq. (17) becomes:

(18) $$\frac{dC_{i}}{dt}=-Da_{0}{C_{i}}^{n}$$

where $Da_{0}$ represents the constant value of the Damköhler number at the initial time.

## 3 Results and discussion

### 3.1 Conversion of a CSTR per pass

**Figure 3:**
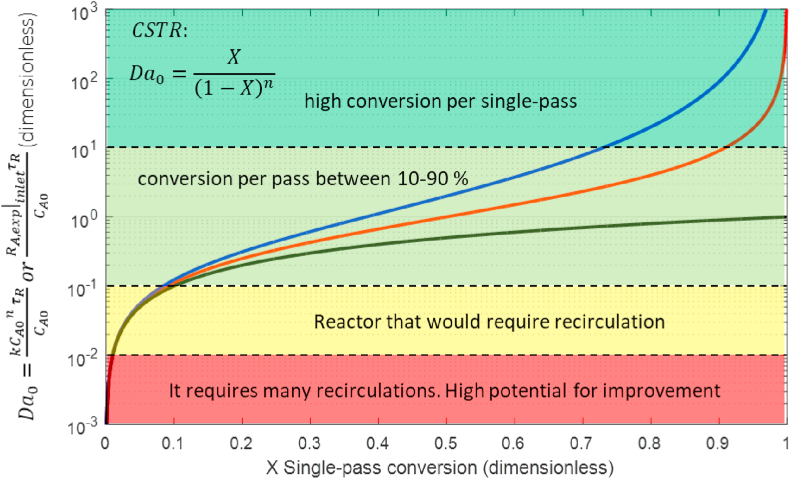
and conversion per pass in a CSTR with n-th power law kinetic rate. Notes: n = 0 black line; n = 1 red line; n = 2 blue line.

Figure 3 shows the relationship between the Da at the CSTR inlet and the conversion at the outlet for a kinetic that follows an n-th power law, steady state, and constant volumetric flow. This figure is generated from Eq. (15) for Da between 10^−3^ and 10^3^.

Figure 3 quickly estimates the single-pass conversion (X) from CSTR inlet variables ($Da_{0}$), where 4 intervals can be identified: (i) $Da_{0}$ < 10^−2^ has X less than 1% and the fluid will require many recirculations to achieve the desired conversion; (ii) 10^−2^ < $Da_{0}$< 10^−1^ presents X less than 10%, the reactor may require some recirculation or an increase in mean residence time to achieve a higher overall conversion; (iii) 10^−1^ < $Da_{0}$< 10 presents X between 10 and 90%, this is known as the rule-of-thumb Damköhler numbers [20], for zero-order kinetic it reaches up to 100% when Da is 1; (iv) $Da_{0}$> 10 shows a high X, the desired conversion could be reached before leaving the reactor, in this case the reactor is oversized.

### 3.2 Design of a CSTR

The CSTR design volume for a desired conversion is calculated by reading $Da_{0}$in Figure 3, and solving Eq. (7) for the volume:

(19) $$V_{CSTR}=\frac{\dot{Q}Da_{0}}{k{C_{A0}}^{n-1}}$$

For example, a conversion of 0.5 is desired, in a first-order reaction with k =10^−6^ s^−1^ and volumetric flow of 1 cm^3^ s^−1^. From Figure 3, with X=0.5 and n=1, we read $Da_{0}$ =1, and by Eq. (19) we calculate the volume of the reactor $V_{CSTR}=10^{6}\;cm^{3}=1\;m^{3}$.

The methodology is simple and useful. Alternatively, without using the graph, we can calculate it as:

(20) $$V_{CSTR}=\frac{\dot{Q}X}{k{C_{A0}}^{n-1}{\left(1-X\right)}^{n}}$$

This result is consistent with the design equation presented in the engineering literature of chemical reactions [20], where $V_{CSTR}=F_{A0}X/{\left(R_{A}\right)}_{outlet}$.

Figure 3 can be used to estimate the conversion if the kinetic parameters are known. For example, the oxidation of As (III) was evaluated in a NETmix photocatalytic mili-reactor [29]. The authors report first-order kinetic constants (4.828, 5.307, 7.907) x10^−4^ s^−1^ for three radiation intensities. The reactor operated for 2 h (τ = 7200 s). The $Da_{0}$ is calculated: $Da_{0}=k\tau =$3.48, 3.82 and 5.69 respectively, and the reading of Figure 3 gives the following conversions 0.78, 0.79, and 0.85 respectively. The authors report experimental conversions of 0.82, 0.84, and 0.90. The error of the proposed graphic methodology is less than 6%, and it is due to the deviation of the experimental data to the first-order model. The reactor (1.5 L) can be scale-up, keeping the $Da_{0}$ constant, which will guarantee the conversion according to Figure 3. The new distribution of light in a larger volume must ensure the order of magnitude of k and the mixing.

### 3.3 Conversion of unidirectional flow reactor per pass

**Figure 4:**
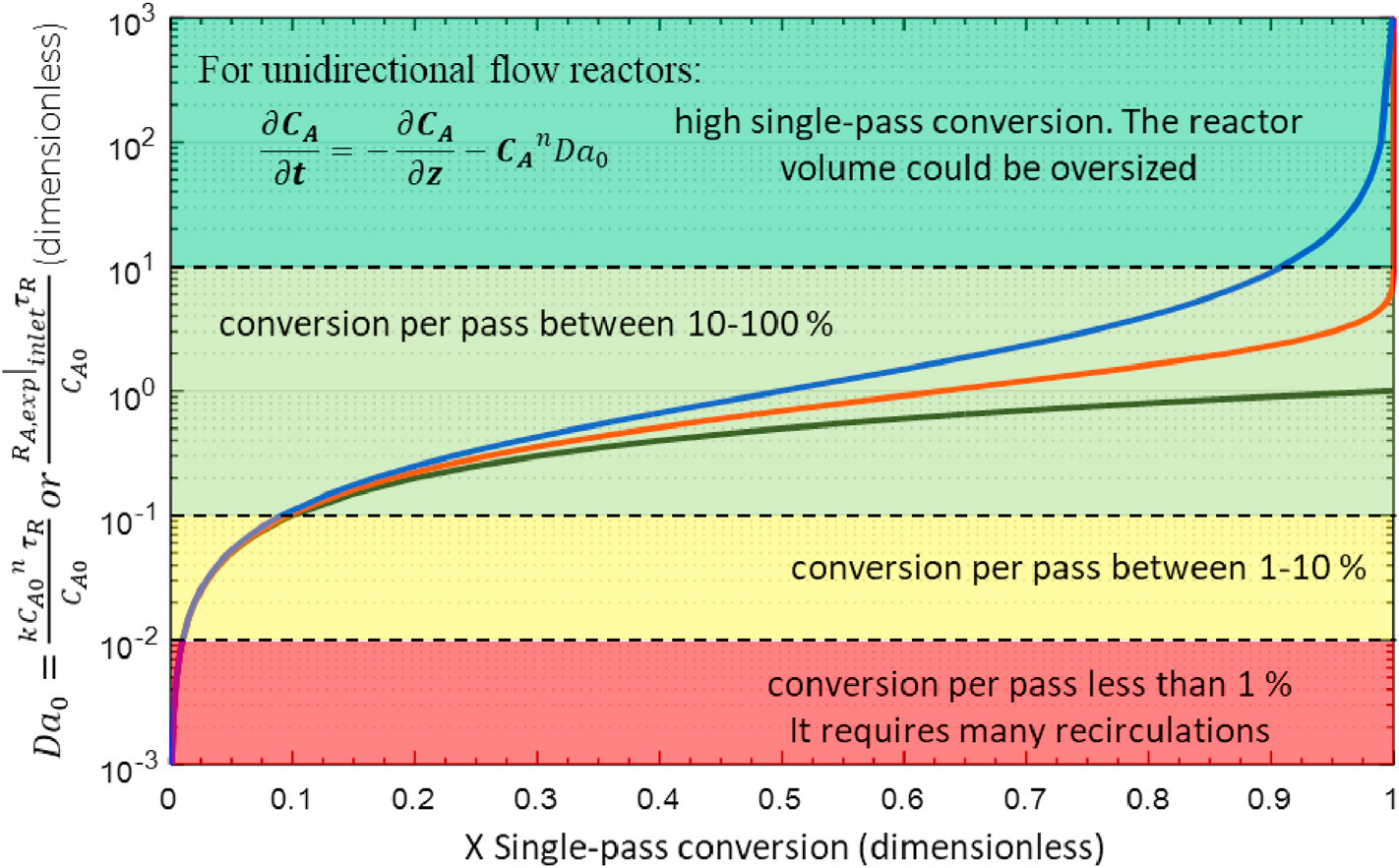
$Da_{0}$ and single-pass conversion (X) at unidirectional flow with n-th order power reaction rate. Notes: n = 0 black line; n = 1 red line; n = 2 blue line.

Figure 4 shows $Da_{0}$as a function of X for the transient state of unidirectional flow reactors that follow reaction rates given by the n-th power law. To obtain these profiles, the PDE of a unidirectional flow reactor (Eq. (11)) is solved by using the orthogonal collocation method coupled to fourth-order Runge Kuta [7, 30]. The solution is a matrix of concentrations of A in all z-coordinate and time, the concentration at the reactor outlet is selected in a time equal to the mean residence time (Conditions: **t**=1, **z**=1, boundary condition III), and the conversion is estimated. The process for $Da_{0}$from 10^−3^ to 10^3^ is repeated. Finally, $Da_{0}$and conversion are plotted.

Figure 4 presents the four intervals of $Da_{0}$for longitudinal flow reactors; each interval has the same interpretation discussed for CSTR reactors. Then, the parameter $Da_{0}$is applied to both unidirectional and isotropic continuous reactors to estimate the conversion per pass quickly.

### 3.4 Design of unidirectional flow reactor: graphical method

For a conversion required (X), $Da_{0}$is determined using Figure 4. The design volume of a unidirectional flow reactor $V_{UFR}$is cleared of the definition of $Da_{0}$. There are different ways of expressing it, all of them consistent with Eq. (19):

(21) $$V_{UFR}=\frac{\dot{Q}Da_{0}}{k{C_{A0}}^{n-1}}=\frac{\dot{Q}C_{A0}Da_{0}}{{R_{A}|}_{inlet}}=\frac{F_{A0}Da_{0}}{{R_{A}|}_{inlet}}$$

For the previous example, the same reaction occurs in a unidirectional flow reactor. It is desired to know the design volume of the new reactor and proceeds as follows. From Figure 4, with X=0.5 and n=1, we read $Da_{0}$= 0.7, and by Eq. (21), we calculate the reactor volume $V_{UFR}=0.7x10^{6}\;cm^{3}=0.7\;m^{3}$. Just 70% compared with the $V_{CSTR}$. This graphical methodology (Fig. (4)) is easy and useful since it avoids the analytical or numerical solution of the partial differential equation of the continuity equation that governs this reactor. Therefore, this proposes a new methodology to design continuous unidirectional flow reactors.

Let us consider another example, for a second-order reaction and required conversion of 50%, then, $Da_{0}$= 1 according to Figure 4, the design volume of the reactor can be calculated as a function of operating flow as follows:

(22) $$V_{design}=\frac{\dot{Q}\left(1\right)}{kC_{A0}}$$

Or experimentally like,

(23) $$V_{design}=\frac{\dot{Q}C_{A0}\left(1\right)}{{R_{A,exp}|}_{inlet}}$$

If the same reaction and conditions now require conversion of 80%, we have from Figure 4 that $Da_{0}$ = 4,

(24) $$V_{design}=\frac{\dot{Q}\left(4\right)}{kC_{A0}}$$

The design volume is four times the previous one.

The design volume of unidirectional flow reactors does not have a simple mathematical relationship with the conversion. However, the design volume is linear with the $Da_{0}$(Eq. (21)). This is very convenient for the design and scaling-up of reactors; e.g., an increase of one (1) magnitude order of $Da_{0}$ leads to an increase of 10 times the reactor volume keeping the other process variables constant. The conversion is determined using Figure 4.

A given conversion is reached at a specific value of $Da_{0}$. Therefore, if different reactors produce the same conversion, they are all operated at the same $Da_{0}$regardless of whether it is a laboratory, bench, pilot, or industrial scale. This result applies to both CSTR and unidirectional flow reactors. Hence, the powerful utility of the $Da_{0}$vs X diagram for scaling-up.

### 3.5 Low Da effect

For $Da_{0}$ < 10^−2^, the single-pass conversion is less than 1% for any kinetic rate. Da ≈ X can be assumed with an error ≤2%. Therefore, these reactors require a high number of recirculations.

Figures 3 and 4 are useful for determining the single-pass conversion from $Da_{0}$into continuous reactors without the need to solve the mathematical models. However, reactors with a low $Da_{0}$, particularly $Da_{0}$ < 0.1, have a low conversion per pass, and they require multiple recirculations.

### 3.6 Multiple passes through the reactor

**Figure 5:**
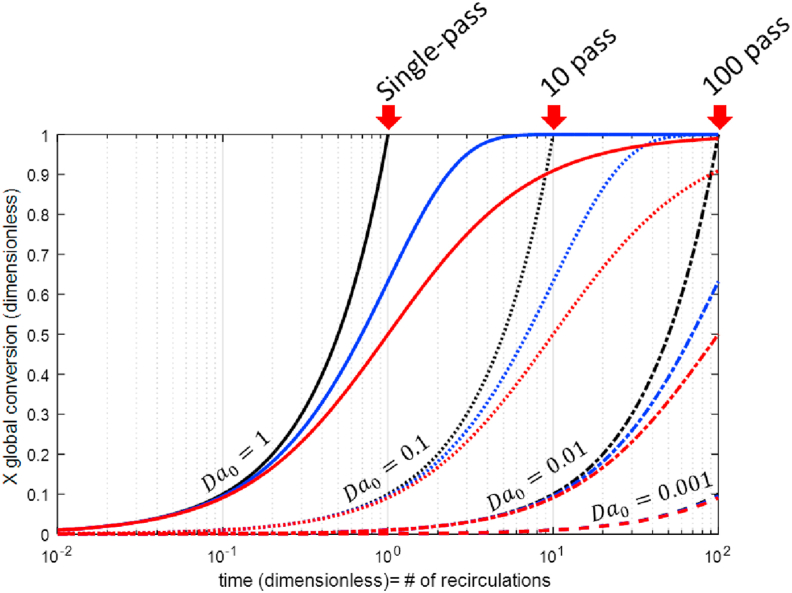
Overall conversion of unidirectional flow reactors in recirculation mode as a function of time and $Da_{0}$with an n-th order power reaction rates and constant volumetric flow. Notes: n = 0 black line; n = 1 blue line; n = 2 red line.

Figure 5 shows the increase of overall conversion as a function of dimensionless time $t=t/\tau_{R}$when the fluid is recirculated through a unidirectional flow reactor. If we define the number of recirculations as the total time between $\tau_{R}$, then t is equal to the number of recirculations.

The black, blue, and red lines correspond to kinetic rates of zero, first and second-order respectively, evaluated for $Da_{0}$from 10^−3^ to 1. This graph was constructed with the procedure of the previous figure. The partial differential equation was solved for tfrom 0 to 100 to evaluate from 1 to 100 recirculations (Conditions: **z** =1, boundary condition III).

Figure 5 is useful for estimating the number of recirculations required to achieve a desired conversion as a function of $Da_{0}$. Systems with 0.1 > $Da_{0}$> 0.001 will require between 10 to 1000 recirculations to achieve conversions higher than 90%, e.g., for $Da_{0}$= 1 × 10^−2^ and second-order reaction, the reactor has a conversion per pass of 0.99%, and for a desired overall conversion of 50%, it will require 100 recirculations.

## 4 Conclusions

A new interpretation of the Damköhler number as a local property Da(t, x, y, z) was proposed to develop scale-up and design tools capable of relating the conversion at the output of a reactor with its properties at the input.

The expression for $Da_{0}$includes the operating conditions as volumetric flow, design volume, or combinations of the above (residence time) and intrinsic reaction rate. This facilitates the rigorous analysis of the reactors, varying only one parameter.

The proposed graphical methodology avoids the analytical or numerical solution of the partial differential equation of continuity that governs the unidirectional flow reactors. This facilitates the design of the reactor volume and scaling-up.

The design volume of reactors does not have a simple mathematical relationship with the conversion. However, it was concluded that the design volume is linear with the Danumber evaluated at the inlet ($Da_{0}$) for both reactors types, and the conversion was obtained from the charts.

A given $Da_{0}$corresponds to one conversion, therefore two reactors of different scale and operating conditions that have the same $Da_{0}$will have the same conversion, this is the key to scale-up reactors.

The reactors with $Da_{0}$< 0.1, have a low conversion per pass and multiple recirculations are required. The charts also facilitate the estimation of the number of recirculations required in reactors with low $Da_{0}$to achieve a desired overall conversion.

## Declarations

### Author contribution statement

Héctor L. Otálvaro-Marín: Conceived and designed the experiments; Performed the experiments; Analyzed and interpreted the data; Contributed reagents, materials, analysis tools or data; Wrote the paper.

Fiderman Machuca-Martínez: Conceived and designed the experiments; Analyzed and interpreted the data; Wrote the paper.

### Funding statement

This work was supported by Universidad del Valle & Colciencias (811-2018): Diseño y simulación, construcción y evaluación de reactores fotocatalíticos heterogéneos de alta eficiencia para tratamiento de aguas residuales basado en los principios físicos y análisis adimensional.

### Competing interest statement

The authors declare no conflict of interest.

### Additional information

No additional information is available for this paper.

## Appendix A Supplementary data

Support information _spl_Heliyon_spl_ Support information _spl_Heliyon_spl_
