# Supplementary material for: Sizing of reactors by charts of Damköhler's number for solutions of dimensionless design equations
Source: Heliyon. 2020 Nov 2;6(11):e05386. doi: 10.1016/j.heliyon.2020.e05386 (PMC7644903; doi:10.1016/j.heliyon.2020.e05386)
Supplement: Support information _spl_Heliyon_spl_ [file mmc1.docx]

**Supporting Information**

**Sizing of reactors by chart of Damköhler's number for solutions of dimensionless design equations**

Héctor L. Otálvaro-Marín^a,b,c*^, Fiderman Machuca-Martinez^a^

^a^ GAOX, Escuela de Ingeniería Química, Universidad del Valle, A.A. 25360, Cali, Colombia. Email: hector.otalvaro@correounivalle.edu.co, fiderman.machuca@correounivalle.edu.co

^b^ MADE Group, Food Engineering Program, Universidad de la Amazonia, Florencia, Colombia.

^c^ IDEI Group, I+D Educación e Ingeniería, Cali, Colombia.

*Corresponding author, email address: [hector.otalvaro@correounivalle.edu.co](mailto:hector.otalvaro@correounivalle.edu.co), fiderman.machuca@correounivalle.edu.co

**Content**

# S1: Derivation of the continuity equation as a dimensionless partial differential equation expressed as a function of Damkohler and Peclet numbers

# S2: Continuity equation as Damköhler function for kinetic rates described by the power law

# S1: Derivation of the continuity equation as a dimensionless partial differential equation expressed as a function of Damkohler and Peclet numbers

Starting from a volume differential element (∆𝑥, ∆𝑦, ∆z), the time-dependent continuity equation is derived for a system where: mass transport due to the global movement of the fluid, mass transport due the diffusion, and formation or consumption of a component due to the reaction rate occur simultaneously (Fig. S1.1). Molar bases have been selected for all terms.

$$x$$

$$z$$

$$y$$

$$N_{\left. i,x \right|x}$$

$$N_{\left. i,x \right|x+\Delta x}$$

$$N_{\left. i,y \right|y+\Delta y}$$

$$N_{\left. i,z \right|z+\Delta z}$$

$$N_{\left. i,y \right|y}$$

$$N_{\left. i,z \right|z}$$

$$N_{i,\xi}=J_{i,\xi}+v_{\xi}C_{i}$$

$$\left( \begin{aligned} molar \\ accumulation \\ rate of \\ component i \end{aligned} \right)=\left( \begin{aligned} Molar \\ flow of \\ component i \\ at the inlet \end{aligned} \right)-\left( \begin{aligned} Molar \\ flow of \\ component i \\ at the outlet \end{aligned} \right)+\left( \begin{aligned} Consumption or \\ formation molar \\ rate of component i \\ by chemical reaction \end{aligned} \right)$$

Fig. S1.1. Volume differential element for continuous mass transfer systems with chemical reaction.

The mass balance (molar) for component $i$ is expressed as:

$\left( \Delta x\Delta y\Delta z \right)\frac{\Delta C_{i}}{\Delta t}=\Delta y\Delta z\left( N_{\left. i,x \right|x}-N_{\left. i,x \right|x+\Delta x} \right)+\Delta x\Delta z\left( N_{\left. i,y \right|y}-N_{\left. i,y \right|y+\Delta y} \right)+\Delta x\Delta y\left( N_{\left. i,z \right|z}-N_{\left. i,z \right|z+\Delta z} \right)+\left( \Delta x\Delta y\Delta z \right)R_{i}$ (S1.1)

Dividing both sides of the equation by $\Delta x\Delta y\Delta z$, and evaluating the limit when $\Delta t\to0$, $\Delta x\to0$, $\Delta y\to0$, and $\Delta z\to0$, leads to the following equivalent expressions:

| $\frac{\partial C_{i}}{\partial t}+\left( \frac{\partial N_{i,x}}{\partial x}+\frac{\partial N_{i,y}}{\partial y}+\frac{\partial N_{i,z}}{\partial z} \right)=R_{i}$ | (S1.2) |
| --- | --- |
| $\frac{\partial C_{i}}{\partial t}+\left( \boldsymbol{\nabla}\boldsymbol{\cdot}{\vec{\boldsymbol{N}}}_{\boldsymbol{i}} \right)=R_{i}$ | (S1.3) |

The parentheses are used to denote that the content is a scalar, and the bold characters are vectors. By definition the relative molar flux density is:

| ${\vec{\boldsymbol{J}^{\boldsymbol{*}}}}_{\boldsymbol{i}}\equiv C_{i}\left( {\vec{\boldsymbol{v}}}_{\boldsymbol{i}}\boldsymbol{-}\vec{\boldsymbol{v}^{\boldsymbol{*}}} \right)$ | (S1.4) |
| --- | --- |

where $\left( {\vec{\boldsymbol{v}}}_{\boldsymbol{i}}\boldsymbol{-}\vec{\boldsymbol{v}^{\boldsymbol{*}}} \right)$ is the $i$-component diffusion velocity relative to the local molar mean velocity ($\vec{\boldsymbol{v}^{\boldsymbol{*}}}$).

| $\vec{\boldsymbol{v}^{\boldsymbol{*}}}\equiv\frac{\sum_{i=1}^{N} C_{i}{\vec{\boldsymbol{v}}}_{\boldsymbol{i}}}{\sum_{i=1}^{N} C_{i}}$ | (S1.5) |
| --- | --- |

Note that $c\vec{\boldsymbol{v}^{\boldsymbol{*}}}$ is the local rate with which the moles pass through a section of area placed perpendicular to $\vec{\boldsymbol{v}^{\boldsymbol{*}}}$. Eq. S1.4 can be rewritten as follows,

| $C_{i}{\vec{\boldsymbol{v}}}_{\boldsymbol{i}}\boldsymbol{=}{\vec{\boldsymbol{J}^{\boldsymbol{*}}}}_{\boldsymbol{i}}+C_{i}\vec{\boldsymbol{v}^{\boldsymbol{*}}}$ | (S1.6) |
| --- | --- |

By definition, the molar flux density of $i$-component relative to stationary coordinates is:

| $\boldsymbol{N}_{\boldsymbol{i}}\equiv C_{i}{\vec{\boldsymbol{v}}}_{\boldsymbol{i}}$ | (S1.7) |
| --- | --- |

So equation S1.6 becomes:

| $N_{i}\boldsymbol{=}{\vec{\boldsymbol{J}^{\boldsymbol{*}}}}_{\boldsymbol{i}}+C_{i}\vec{\boldsymbol{v}^{\boldsymbol{*}}}$ | (S1.8) |
| --- | --- |

The molar flow density of $i$-component is due to the contribution of two vectors: the diffusion molar flow density of $i$ and the molar flow density of $i$ due to the global movement of the fluid.

The diffusion of $i$ is the mass transport due to a concentration gradient of $i$ in the mixture and the global movement of the fluid is the mass transport due to differences in pressure (advection), temperatures and densities (convection), tangential forces ( viscous), or other forces.

Replacing $N_{i}$ (Ec. S1.8) in Ec. (S1.3), and operating the gradient on each contribution:

| $\frac{\partial C_{i}}{\partial t}+\left( \boldsymbol{\nabla}\boldsymbol{\cdot}{\vec{\boldsymbol{J}^{\boldsymbol{*}}}}_{\boldsymbol{i}} \right)\boldsymbol{+}\left( \boldsymbol{\nabla}\boldsymbol{\cdot}C_{i}\vec{\boldsymbol{v}^{\boldsymbol{*}}} \right)=R_{i}$ | (S1.9) |
| --- | --- |

The Fick's first law on a molar basis is:

| ${\vec{\boldsymbol{J}^{\boldsymbol{*}}}}_{\boldsymbol{i}}\boldsymbol{=-}C\mathcal{D}\boldsymbol{\nabla}x_{i}$ | (S1.10) |
| --- | --- |

where $C$ is the concentration of the mixture and $\mathcal{D}$ is the diffusivity of $i$ in the mixture. Replacing,

| $\frac{\partial C_{i}}{\partial t}-\left( \boldsymbol{\nabla}\boldsymbol{\cdot}C\mathcal{D}\boldsymbol{\nabla}x_{i} \right)\boldsymbol{+}\left( \boldsymbol{\nabla}\boldsymbol{\cdot}C_{i}\vec{\boldsymbol{v}^{\boldsymbol{*}}} \right)=R_{i}$ | (S1.11) |
| --- | --- |

This equation is valid for systems of variable total density ($\rho or C$), and variable diffusivity $\mathcal{D}$.

Assuming the molar concentration $C$ of the mixture and the coefficient of diffusivity constant, the previous expression becomes:

| $\frac{\partial C_{i}}{\partial t}-\mathcal{D}\left( \boldsymbol{\nabla}^{\boldsymbol{2}}C_{i} \right)\boldsymbol{+}\left( \boldsymbol{\nabla}\boldsymbol{\cdot}C_{i}\vec{\boldsymbol{v}^{\boldsymbol{*}}} \right)=R_{i}$ | (S1.12) |
| --- | --- |

Let's analyze the global movement of the fluid $\left( \boldsymbol{\nabla}\boldsymbol{\cdot}C_{i}\vec{\boldsymbol{v}^{\boldsymbol{*}}} \right)$. For practical engineering purposes the average velocity (in a cross section of flow) of the overall movement of the fluid is $\bar{\text{v}}$, which is estimated as:

| $\bar{\text{v}}=\frac{\iint\text{v}\left( \xi_{1},\xi_{1},\xi_{1} \right)dA}{\iint dA}$ | (S1.13) |
| --- | --- |

Or experimentally,

| $\bar{\text{v}}=\frac{volumetric flow}{Area}$ | (S1.14) |
| --- | --- |

where $\text{v}$ does not discriminate between $\vec{\boldsymbol{v}^{\boldsymbol{*}}}$ and $\boldsymbol{v}$. We can conclude that in engineering practice, $\text{v}\boldsymbol{=v=}\vec{\boldsymbol{v}^{\boldsymbol{*}}}$ for mixing, regardless of the individual velocity of the species, and the (mass or molar) base.

For a incompressible fluid (fluid of constant density), $\left( \boldsymbol{\nabla}\boldsymbol{\cdot v} \right)\boldsymbol{=}0$, then,

| $\left( \boldsymbol{\nabla}\boldsymbol{\cdot}C_{i}\boldsymbol{v} \right)\boldsymbol{=}C_{i}\left( \boldsymbol{\nabla}\boldsymbol{\cdot}\boldsymbol{v} \right)\boldsymbol{+}\left( \boldsymbol{v}\boldsymbol{\cdot}\boldsymbol{\nabla}C_{i} \right)\boldsymbol{=}\left( \boldsymbol{v}\boldsymbol{\cdot}\boldsymbol{\nabla}C_{i} \right)$ | (S1.15) |
| --- | --- |

Replacing in the continuity equation, the expression to determine concentration profiles of $i$-component in systems dependent on time with diffusion, advection, chemical reaction, under conditions of density and diffusivity coefficient constant is obtained:

| $\frac{\partial C_{i}}{\partial t}-\mathcal{D}\left( \boldsymbol{\nabla}^{\boldsymbol{2}}C_{i} \right)\boldsymbol{+}\left( \boldsymbol{v}\boldsymbol{\cdot}\boldsymbol{\nabla}C_{i} \right)=R_{i}$ | (S1.16) |
| --- | --- |

The Eq. shows how $D$, $\boldsymbol{v}$, and $R_{i}$ are related in the material balance.

Let us assume a one-direction flow at average velocity, $v=\left\langle v_{z} \right\rangle$. So,

| $\frac{\partial C_{i}}{\partial t}-\mathcal{D}\frac{\partial^{2}C_{i}}{\partial z^{2}}\boldsymbol{+}v\frac{\partial C_{i}}{\partial z}=R_{i}$ | (S1.17) |
| --- | --- |

Now, the following expressions are defined:

| $\boldsymbol{C}_{\boldsymbol{i}}{\equiv C_{i}}/{{C_{i}}^{0}}$ | (S1.18) |
| --- | --- |
| $\boldsymbol{z}\equiv z/{L_{R}}$ | (S1.19) |
| $\boldsymbol{t}\equiv t/{\tau_{R}}$ | (S1.20) |

where the bold variables are dimensionless; ${C_{i}}^{0}$ it is the initial concentration; $L_{R}$ is the reactor length; $\tau_{R}$ is the mean residence time at the reactor.

The variables $C_{i}$, $z$, $t$ are solved according to Eqs. (S1.18-S1.20); the derivatives of Eq. (S1.17) are evaluated which are functions of ${C_{i}}^{0}$, $L_{R}$, $\tau_{R}$ and $\boldsymbol{C}_{\boldsymbol{i}}$, $\boldsymbol{z}$, $\boldsymbol{t}$. The derivatives at Eq. (S1.17) are replaced:

| $\frac{{C_{i}}^{0}}{\tau_{R}}\frac{\partial\boldsymbol{C}_{\boldsymbol{i}}}{\partial\boldsymbol{t}}-D\frac{{C_{i}}^{0}}{{L_{R}}^{2}}\frac{\partial^{2}\boldsymbol{C}_{\boldsymbol{i}}}{{\partial\boldsymbol{z}}^{2}}+v\frac{{C_{i}}^{0}}{L_{R}}\frac{\partial\boldsymbol{C}_{\boldsymbol{i}}}{\partial\boldsymbol{z}}=R_{i}$ | (S1.21) |
| --- | --- |

The first term is isolated:

| $\frac{\partial\boldsymbol{C}_{\boldsymbol{i}}}{\partial\boldsymbol{t}}=D\frac{\tau_{R}}{{L_{R}}^{2}}\frac{\partial^{2}\boldsymbol{C}_{\boldsymbol{i}}}{{\partial\boldsymbol{z}}^{2}}-v\frac{\tau_{R}}{L_{R}}\frac{\partial\boldsymbol{C}_{\boldsymbol{i}}}{\partial\boldsymbol{z}}+R_{i}\frac{\tau_{R}}{{C_{i}}^{0}}$ | (S1.22) |
| --- | --- |

The mean residence time in a longitudinal flow reactor is ${L_{R}}/v$, therefore, $v\frac{\tau_{R}}{L_{R}}$ is equal to 1. The expressions that define Da and Pe are deducted from Eq. (S1.22):

| $Da\equiv-R_{i}\frac{\tau_{R}}{{C_{i}}^{0}}$ | (S1.23) |
| --- | --- |
| $Pe\equiv{{L_{R}}^{2}}/\left( D\tau_{R} \right)$ | (S1.24) |

The negative sign in Eq. (S1.23) was conveniently chosen to express the positive Da on mineralization reactions. We can rewrite the mass balance, Eq. (S1.22), with dimensionless variables:

| $\frac{\partial\boldsymbol{C}_{\boldsymbol{i}}}{\partial\boldsymbol{t}}=\frac{1}{Pe}\frac{\partial^{2}\boldsymbol{C}_{\boldsymbol{i}}}{{\partial\boldsymbol{z}}^{2}}-\frac{\partial\boldsymbol{C}_{\boldsymbol{i}}}{\partial\boldsymbol{z}}-Da$ | (S1.25) |
| --- | --- |

Eq. (S1.25) is the continuity equation of $i$ as function of dimensionless variables applied for any function of the reaction rate, $Da=f\left( t,z \right)$, and $Pe$.

# S2: Continuity equation as Damköhler function for kinetic rates described by the power law

Let’s consider a reaction that follows an n-th power reaction rate,

| $-R_{i}=k{C_{i}}^{n}$ | (S2.1) |
| --- | --- |

where $k$ is the kinetic constant. Also, the concentration as a function of the conversion is expressed as follows:

| $C_{i}={C_{i}}^{0}(1-X)$ | (S2.2) |
| --- | --- |

where ${C_{i}}^{0}$ is the initial concentration and equal to the reactor inlet concentration $C_{i0}$. Now, Eq. (S2.2) is replaced in the reaction rate to obtain:

| ${-R}_{A}=k{{C_{i0}}^{n}(1-X)}^{n}$ | (S2.3) |
| --- | --- |

So, the Eq. (S1.23) is as follows:

| $Da={(1-X)}^{n}\left( k{C_{i0}}^{n}\frac{\tau_{R}}{C_{i0}} \right)$ | (S2.4) |
| --- | --- |

Note that the second factor on the right side is the $Da$ number evaluated at the reactor inlet (Eq. S1.23). We call ${Da}_{0}$ to:

| ${Da}_{0}\equiv\frac{k{C_{i0}}^{n} \tau_{R}}{C_{i0}}$ | (S2.5) |
| --- | --- |

The Damköhler number ($Da$) is usually defined in the above way in the literature. However, there are differences between the $Da$ at any point and time and the ${Da}_{0}$ at entry.

Therefore, for a system that follows a reaction rate of n-power law we have to:

| $Da={(1-X)}^{n}{Da}_{0}$ | (S2.6) |
| --- | --- |

This equation relates the $Da$ at any point (at z-coordinate) and any time within the reactor to the conversion and the inlet properties. For $n$ = 0, $Da$ is constant in all space and equal to its value at the inlet.

From Eqs. (S1.18) and (S2.2) we obtain:

| $\boldsymbol{C}_{\boldsymbol{i}}=\frac{C_{i}}{C_{i0}}=(1-X)$ | (S2.7) |
| --- | --- |

Substituting Eq. S2.7 into S2.6,

| $Da={\boldsymbol{C}_{\boldsymbol{i}}}^{n}{Da}_{0}$ | (S2.8) |
| --- | --- |

Substituting $Da$ at Eq. (S1.25),

| $\frac{\partial\boldsymbol{C}_{\boldsymbol{i}}}{\partial\boldsymbol{t}}=\frac{1}{Pe}\frac{\partial^{2}\boldsymbol{C}_{\boldsymbol{i}}}{{\partial\boldsymbol{z}}^{2}}-\frac{\partial\boldsymbol{C}_{\boldsymbol{i}}}{\partial\boldsymbol{z}}-{\boldsymbol{C}_{\boldsymbol{i}}}^{n}{Da}_{0}$ | (S2.9) |
| --- | --- |

Eq. (S2.9) is specifically for systems that obey a reaction rate expressed by power law. ${Da}_{0}$ can be a constant value or function of time, and this equation can be solved to determine the conversion (or $\boldsymbol{C}_{\boldsymbol{i}}$) at the reactor outlet as a function of ${Da}_{0}$.

If advection is the dominant mass transport, then, $\frac{1}{Pe}\approx0$, the Eq. (S2.9) becomes

| $\frac{\partial\boldsymbol{C}_{\boldsymbol{i}}}{\partial\boldsymbol{t}}=-\frac{\partial\boldsymbol{C}_{\boldsymbol{i}}}{\partial\boldsymbol{z}}-{\boldsymbol{C}_{\boldsymbol{i}}}^{n}{Da}_{0}$ | (S2.10) |
| --- | --- |
